# Effectiveness of Human-Supported and Self-Help eHealth Lifestyle Interventions for Patients With Cardiometabolic Risk Factors: A Meta-Analysis

**DOI:** 10.1097/PSY.0000000000001242

**Published:** 2023-10-09

**Authors:** Talia R. Cohen Rodrigues, Linda D. Breeman, Asena Kinik, Thomas Reijnders, Elise Dusseldorp, Veronica R. Janssen, Roderik A. Kraaijenhagen, Douwe E. Atsma, Andrea W.M. Evers

**Affiliations:** From the Health, Medical, and Neuropsychology Unit, Institute of Psychology (Cohen Rodrigues, Breeman, Kinik, Reijnders, Janssen, Evers), and Institute of Psychology, Methodology and Statistics Group (Dusseldorp), Leiden University; Department of Cardiology (Janssen, Atsma), Leiden University Medical Center, Leiden; NDDO Institute for Prevention and Early Diagnostics (NIPED) (Kraaijenhagen), Amsterdam; Vital10 (Kraaijenhagen), Amsterdam; Department of Psychiatry (Evers), Leiden University Medical Center, Leiden; and Medical Delta, Leiden University, Technical University of Delft, and Erasmus University Rotterdam (Evers), the Netherlands.

**Keywords:** cardiovascular disease, chronic kidney disease, type 1 diabetes mellitus, type 2 diabetes mellitus, eHealth, lifestyle change, human support, **CKD** = chronic kidney disease, **CVD** = cardiovascular disease, **T1DM** = type 1 diabetes mellitus, **T2DM** = type 2 diabetes mellitus

## Abstract

**Objective:**

eHealth is a useful tool to deliver lifestyle interventions for patients with cardiometabolic diseases. However, there are inconsistent findings about whether these eHealth interventions should be supported by a human professional, or whether self-help interventions are equally effective.

**Methods:**

Databases were searched between January 1995 and October 2021 for randomized controlled trials on cardiometabolic diseases (cardiovascular disease, chronic kidney disease, type 1 and 2 diabetes mellitus) and eHealth lifestyle interventions. A multilevel meta-analysis was used to pool clinical and behavioral health outcomes. Moderator analyses assessed the effect of intervention type (self-help versus human-supported), dose of human support (minor versus major part of intervention), and delivery mode of human support (remote versus blended). One hundred seven articles fulfilled eligibility criteria and 102 unique (*N* = 20,781) studies were included.

**Results:**

The analysis showed a positive effect of eHealth lifestyle interventions on clinical and behavioral health outcomes (*p* < .001). However, these effects were not moderated by intervention type (*p* = .169), dose (*p* = .698), or delivery mode of human support (*p* = .557).

**Conclusions:**

This shows that self-help eHealth interventions are equally effective as human-supported ones in improving health outcomes among patients with cardiometabolic disease. Future studies could investigate whether higher-quality eHealth interventions compensate for a lack of human support.

**Meta-analysis registration:** PROSPERO CRD42021269263.

## INTRODUCTION

Cardiometabolic diseases, that is, diseases to the heart, are an increasing threat to patients’ health and quality of life (1,2). This includes cardiovascular diseases (CVDs) and type 1 and 2 diabetes mellitus (T1DM and T2DM) and comprises conditions such as chronic kidney disease (CKD). These diseases share similar underlying clinical risk factors, such as adiposity, high blood pressure, cholesterol levels, and blood glucose levels (3,4). Moreover, these four diseases have similar behavioral risk factors, such as smoking, physical inactivity, unhealthy diet, and use of alcohol, which is why a healthy lifestyle is the preferred management strategy for all (3,4). Participating in lifestyle interventions can therefore improve patients’ health and quality of life (5).

Nevertheless, many patients who have participated in cardiac rehabilitation experience difficulties in maintaining a healthy lifestyle in the long term (6). Research suggests that the use of home-based interventions is more suitable for durable lifestyle change compared with traditional face-to-face interventions (7). For that reason, the implementation of eHealth could be beneficial. eHealth can be defined as the use of information and communication technology, such as the Internet, to support or enhance health and health care by means of remote or automated support (8). eHealth lifestyle interventions show to be effective in improving cardiometabolic risk factors. For example, eHealth interventions aimed at physical activity or nutrition can improve clinical risk factors such as blood glucose levels (9) and blood pressure (10), and behavioral risk factors such as fat, fruit and vegetable consumption, and physical activity (11). Another advantage of eHealth over face-to-face interventions is that the former is easier to implement in a larger and more varied audience. Especially self-help interventions are suitable for widespread implementation, as no human care professional needs to be involved (8). Self-help interventions could help reduce the workload for care professionals and the costs of treatment (12). Furthermore, studies show that eHealth interventions with low or even no involvement of care professionals are effective in improving clinical and behavioral risk factors among people with CVD (13).

Despite these advantages, previous meta-analyses and reviews showed mixed results regarding the effect of self-help interventions through eHealth. Notably, some studies have found higher effect sizes for digital interventions in which the feedback was provided by a human (14). This meant that interventions with fully remote human support (15), or those that additionally incorporated face-to-face human support (otherwise called blended interventions) had more effect (i.e., higher effect sizes) than self-help eHealth interventions without any form of human support. In previous studies, authors have argued that human supported interventions are more effective compared with interventions with only automated feedback because they are tailored to the patient’s needs (14). Furthermore, human support is found to increase adherence to interventions (15). In addition, blended interventions would be more effective than fully remote-supported interventions because behavior change maintenance is more successful in when they involve face-to-face interactions (16). In other studies, however, no differences were found in achieving lifestyle behavior change between human-supported and self-help only lifestyle interventions (17), blended interventions compared with remotely-supported ones (18), and interventions with automated feedback compared with those with human-generated feedback (19). These discrepancies in research findings could be explained by the varying “support dose” (e.g., frequency of contact) within the human-supported interventions. Previous meta-analyses regarding eHealth lifestyle interventions have simply categorized studies into self-help or human-supported, or into blended and remote support. In particular, these meta-analyses made no distinction between the type and channel of human support. This meant that studies in which a clinical psychologist gives daily feedback on assignments, studies in which psychology students give monthly telephone calls based on a script, or studies in which patients have the option to contact a therapist were all treated alike. In contrast, various meta-analyses regarding psychological interventions have looked at these variables in more detail. One of these meta-analyses found that interventions with greater amounts of therapeutic contact encountered lower dropout rates (20). Other studies found that both administrative support by a layperson and therapeutic support by a professional are equally effective in treating symptoms and preventing dropout (21,22). Similar results have been found in a meta-analysis regarding digital mental health interventions (23). Other meta-analyses regarding eHealth interventions revealed that higher intensity of support improves intervention adherence rates (24,25).

To our knowledge, no other studies have yet focused on the effectiveness of (human-supported and self-help) eHealth lifestyle interventions for multiple cardiometabolic risk factors, or investigated whether the dose of human support in eHealth lifestyle interventions is related to the effectiveness of these interventions. Therefore, the aims of this meta-analysis are as follows: a) investigating the effectiveness of eHealth lifestyle interventions for people with or at risk of CVD, CKD, T1DM, and T2DM on clinical and behavioral health outcomes; b) investigating whether there is a difference in the effectiveness of human-supported and self-help eHealth lifestyle interventions on clinical and behavioral health outcomes; and c) investigating whether moderating factors such as dose and delivery mode of human support influence the effectiveness of eHealth lifestyle interventions on clinical and behavioral health outcomes.

## METHODS

We preregistered our meta-analysis in the PROSPERO database (PROSPERO 2021 CRD42021269263; (26)). The meta-analysis was conducted in accordance with the Preferred Reporting Items for Systematic Reviews and Meta-Analyses (27).

### Search and Study Selection

**FIGURE 1:**
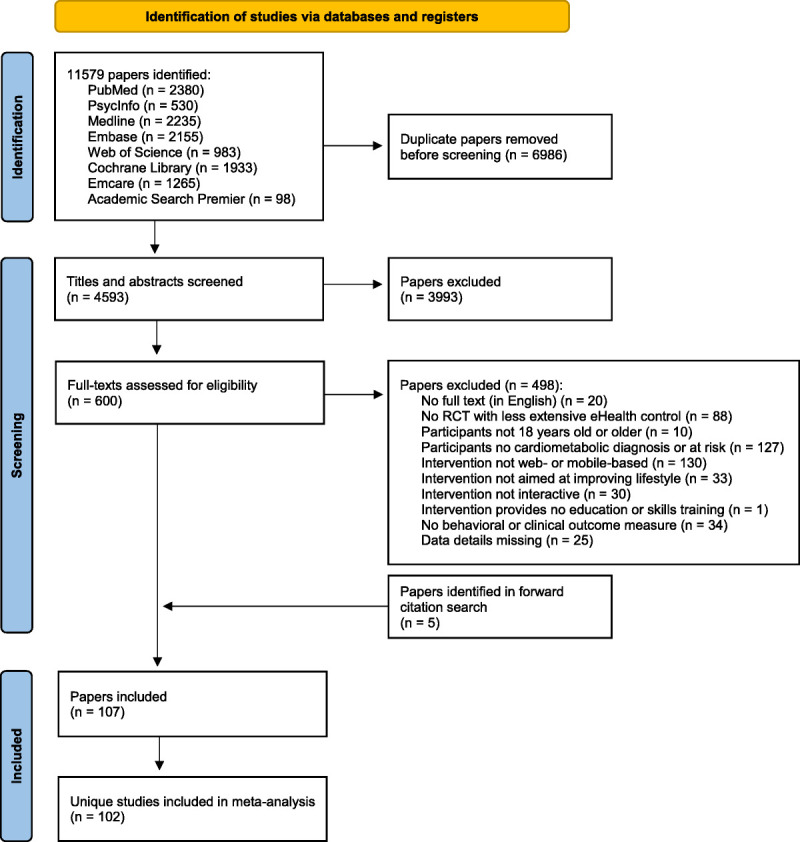
PRISMA flowchart of literature search and screening. PRISMA = Preferred Reporting Items for Systematic Reviews and Meta-Analyses.

A systematic literature search was conducted within multiple databases (Figure 1). With the help of the university’s librarian, a search string was created with key search terms related to a) eHealth, b) clinical and behavioral outcomes, c) cardiometabolic diseases, and d) randomized controlled trials (see the Supplemental Digital Content, http://links.lww.com/PSYMED/A960 for the full search string). The search was conducted for studies from 1995 (given the increasing use of Internet from that year onward) and was lastly updated on October 6, 2021. After removal of duplicates, titles and abstracts were screened by two of the three independent researchers to identify studies meeting the inclusion criteria. Inconsistencies were resolved in weekly discussions.

Inclusion and exclusion criteria were established with the help of the PICO statement (population, intervention, comparator, outcome; (28)). Participants of the included studies were required to a) be 18 years or older and b) either have one or more cardiometabolic risk factors (as determined and specifically mentioned by the authors of the article) or be diagnosed with CVD, CKD, T1DM, or T2DM. Given the primary focus of our study on cardiometabolic patients, we decided to, in case of a population with cardiometabolic risk factors only, exclude studies if cardiometabolic patients were explicitly excluded from participation. Furthermore, studies were included if the intervention c) aimed at improving one or more lifestyle behaviors (physical activity, nutrition, smoking, alcohol intake, sleep), d) was delivered via eHealth tools such as through a website or mobile-based application (phone, text-messages: videoconferencing could be used, but not as main mode of communication), e) provided education or skills training (e.g., using behavior change techniques), and f) was interactive (involving actions of a user and reactions from the program in response to a user’s actions). In addition to this, we only included g) randomized controlled trials, which used as a comparator, either a passive control (wait-list or usual care), a non–web- or mobile-based intervention, or a less extensive web- or mobile-based intervention. Finally, studies were included if h) they reported minimally one self-reported or objectively observed clinical (e.g., blood pressure) or behavioral health outcome (e.g., step count), and i) the full-text was available in English or Dutch. These inclusion and exclusion criteria were used to check for study eligibility, which was again conducted by two of the three independent researchers. Disagreements were resolved in weekly meetings, and if needed, with the help of a third independent researcher. If two articles reported on the same study, we included the one reporting the outcomes most extensively. After the systematic search, we conducted a forward citation search to find relevant articles that either cited one of our included studies or were written by one of the authors of our included studies. Finally, we ran a backward citation search to look at articles cited by the authors included in our study. In case original data were not available in the article, we contacted the relevant authors in writing to ask for the data. Authors were contacted a maximum of two times over a period of 3 months.

### Data Extraction

A predefined coding form was used to extract the data. We extracted a) study characteristics, b) population characteristics at baseline, c) characteristics of each condition (control and intervention), and d) self-reported or objectively observed clinical or behavioral outcome data. For the population characteristics, we coded the diagnosis of the participants (CVD, T1DM, T2DM, CKD, at-risk population [without diagnosis but with cardiometabolic risk factors], or mixed patient population), mean age of the participants per group, percentage of female participants per group, and educational level of the participants per group. For the condition characteristics, we coded the type of control condition (passive or active), intervention length (duration of the intervention in weeks irrespective of pre-post design or longer-term follow-ups), the type of intervention (self-help or human-supported), dose of human support (minor or major part of intervention), and delivery mode of human support (remote or blended). Type of intervention was coded as “self-help” if the study investigated an intervention without any involvement from another human coach and could be followed completely independently, and as “human-supported” if a human coach (health care professional or layperson) was involved to support the participant in following the intervention. Dose of human support was coded as “minor” if the study investigated an intervention that was delivered through an eHealth tool, which the patient could practice independently or with some additional involvement of a human coach. It was coded as “major” if the study investigated an intervention that was delivered by a human coach, in which eHealth served as an additional tool that supported the human guidance. Delivery mode was coded as “remote” if human support was solely delivered via mediated forms of communication (e.g., text messages), and as “blended” if the human support was delivered both via digital communication tools and in face-to-face settings. For the outcome data, all self-reported or objectively observed clinical (blood pressure, glucose, cholesterol, weight, CVD composite score, physical activity capacity) and behavioral (physical activity behavior, smoking, nutrition, alcohol, sleep and relaxation) outcome data were extracted. We decided to treat physical activity capacity, such as distance walked in a specific amount of time or oxygen uptake during physical effort (VO_2max_), as a clinical variable and physical activity behavior, such as steps or minutes of physical activity per day, as a behavioral variable. For each outcome variable, baseline and follow-up measures, mean differences (pre-post measure within one group), or change scores (difference between control and experimental groups) were extracted. In case of multiple intervention conditions, all conditions were extracted, and in case of multiple control conditions, only the least extensive condition was extracted. To assess the methodological quality, we used the latest Cochrane Risk of Bias tool (RoB 2.0) to extract and assess potential risks at the study level regarding the randomization process, deviations from intended interventions (effect of assignment), missing outcome data, measurement of the outcome, and selection of the reported result (29). Studies were assessed as “low,” “some concerns,” or “high” risk of bias in the aforementioned domains. For each study, two of the three independent researchers conducted both the data extraction and risk of bias assessment, and compared their outcomes (interrater reliability of 78%). Possible differences were all resolved in regular meetings, and if needed, with the help of a third independent researcher. Corresponding authors were contacted in case of missing information on key variables.

### Statistical Analyses

An important feature differentiating this study from existing meta-analyses on the effectiveness of eHealth lifestyle interventions for cardiometabolic diseases is our use of a multilevel approach. Rather than conducting a meta-analysis for each outcome separately, a three-level model allowed us to combine different outcome variables from the same study, as it can deal with interdepency of effect sizes (30). The analyses were performed with the Metafor package in RStudio (version 1.4.1103). We estimated pooled effects for all clinical and behavioral outcome variables, using a random-effects multilevel model (30). We used a three-level model to take into account that multiple effect sizes can be nested within a sample. This model allows for effect size variance (level 1), nested in effect sizes (level 2), and nested in study samples (level 3). Thus, all outcomes of each study were included in the analysis and coded with the same study ID. For continuous variables, standardized mean differences (Hedges *g*) with 95% confidence intervals were calculated (31). For categorical variables, we calculated odds ratios with 95% confidence intervals and transformed those to standardized mean differences (32). Variances were calculated based on the provided standard deviations or confidence intervals (33). In case outcomes were measured at multiple time points, we included the outcome directly measured after the end of the intervention as defined by the studies. The intention was to prevent a large variety in long-term measurements.

We assessed publication bias by inspecting funnel plots and performed an Egger test (34) with the Metafor package in RStudio. Publication bias results from studies reporting statistically or clinical significant results more often than nonsignificant results (34). Hence, the effect sizes of studies included in the meta-analysis can differ from the general effect size if all (including nonsignificant) studies would be considered. We determined statistical heterogeneity using log-likelihood ratio tests for both within-study variance (level 2) and between-study variance (level 3) (30). In addition, we conducted moderator analyses to assess the effectiveness of self-help and human-supported eHealth lifestyle interventions, and the effect of dose and delivery mode of human support on the effectiveness of eHealth lifestyle interventions on clinical and behavioral health outcomes. For this, the three-level random-effects model was extended to a three-level mixed-effects model (30) with the following moderators: type of intervention (self-help versus human-supported), dose of human support (minor versus major part of intervention), and delivery mode of human support (remote versus blended). Furthermore, we conducted a moderator analysis with the risk of bias scores (low risk of bias, some concerns, and high risk of bias) and study, intervention, and population characteristics (control condition type, intervention length, patient age, and diagnosis).

## RESULTS

### Study Selection

The search resulted in 4593 articles without duplicates. After abstract screening, a total of 600 full texts were screened for eligibility. Four hundred ninety-eight articles did not meet the eligibility criteria and were therefore excluded. Five more articles were identified during the forward search, which resulted in a total of 107 articles fulfilling the eligibility criteria, corresponding with 102 unique studies. The study selection process is summarized in Figure 1 (27).

### Study Characteristics

The 102 studies produced 809 effect sizes, which all reflected the association between the use of an eHealth lifestyle intervention and either a clinical or behavioral outcome. A total of *N* = 20,781 patients were included in the studies, of which were *n* = 3428 CVD patients (26 studies), *n* = 72 T1DM patients (1 study), *n* = 7.143 T2DM patients (38 studies), *n* = 365 CKD patients (3 studies), *n* = 3648 people at risk (19 studies), and *n* = 6125 patients from a sample with a combination of two or more of the aforementioned diseases (15 studies). Sample sizes ranged from 20 to 2724. The mean age of the patients ranged from 35.2 to 75.9 years. All studies included a combination of female and male patients. The duration of the interventions ranged from 1.5 to 24 months. The majority of the studies investigated the effect of interventions aimed either at physical activity (25) or a combination of multiple lifestyle behaviors (70). 30 investigated interventions (29%) were self-help, while 85 interventions (83%) offered some form of human support. See Table S1, Supplemental Digital Content, http://links.lww.com/PSYMED/A960, for an overview of all studies included in the meta-analysis.

### Risk of Bias Assessment and Publication Bias

The methodological quality of the included studies varied but was overall sufficient. Almost all studies scored “some concerns” on one of the domains in the risk of bias assessment, resulting in a “some concerns” overall score for the majority of the studies (see Table S2, Supplemental Digital Content, http://links.lww.com/PSYMED/A960). We found that the risk of bias score did not moderate the association between eHealth lifestyle interventions and clinical and behavioral health outcomes (*F*(2,829) = 0.637, *p* = .529). This indicates that there were no significant differences in mean effect size between studies with a low risk of bias, some concerns, or high risk of bias score.

Possible publication bias was initially examined by visual inspection of a funnel plot. The funnel plot showed some asymmetry (indicating possible publication bias). Next, we tested funnel plot asymmetry by regressing the standard normal deviation against the estimate’s precision (34). The analysis confirmed the visual inspection of the funnel plot and showed that the intercept significantly deviated from zero (*t*(808) = 3.12, *p* < .001). This means that there are reasons to believe that there is a publication bias for studies on eHealth lifestyle interventions.

### Effectiveness of eHealth Lifestyle Interventions

The overall mean effect size of eHealth lifestyle interventions on clinical and behavioral health outcomes is 0.10 (expressed in Hedges *g*; *p* < .001). A standardized mean difference of 0.10 is considered as small (35). This indicates that patients with cardiometabolic diseases who follow an eHealth lifestyle intervention show more improvement in clinical and behavioral health outcomes compared with patients in control conditions. The overall mean effect sizes of eHealth lifestyle interventions on clinical outcomes only and behavioral outcomes only were 0.09 (*p* < .001) and 0.13 (*p* < .001); Table 1). We did not find a significant difference between the mean effect sizes of eHealth lifestyle interventions on clinical versus behavioral health outcomes (*p* = .051).

**TABLE 1:** Mean Effect Sizes (Expressed in Hedges *g*) for Each Outcome Category

Outcome Category	No. Studies	No. ES	Mean ES (SE)	95% CI	*t* Value	*p*	Within-Study Variance	Between-Study Variance
All outcomes	102	809	0.100 (0.018)	0.065 to 0.135	5.635	<.001***	0.056***	0.014***
Clinical outcomes	92	597	0.086 (0.019)	0.050 to 0.123	4.672	<.001***	0.066***	0.010**
Blood pressure	49	99	0.067 (0.042)	−0.016 to 0.150	1.597	.101	0.034***	0.047***
Glucose	55	84	0.161 (0.069)	0.024 to 0.298	2.343	.022*	0.000	0.220***
Cholesterol	44	157	−0.007 (0.026)	−0.057 to 0.044	−0.270	.788	0.003	0.016***
Weight	60	138	0.117 (0.048)	0.023 to 0.211	2.463	.015*	0.026***	0.098***
CVD composite score	9	11	0.025 (0.031)	−0.044 to 0.095	0.814	.435	0.000	0.000
PA capacity	24	61	0.138 (0.036)	0.065 to 0.211	3.794	<.001***	0.022*	0.000
Behavioral outcomes	60	212	0.131 (0.031)	0.069 to 0.193	4.165	<.001***	0.020***	0.031***
PA behavior	49	119	0.170 (0.038)	0.094 to 0.246	4.453	<.001***	0.000	0.045***
Smoking	11	12	−.086 (0.056)	−0.209 to 0.037	−1.533	.154	0.000	0.013
Nutrition	24	74	0.133 (0.048)	0.037 to 0.229	2.756	.007**	0.040***	0.020*
Alcohol	3	3	−0.085 (0.085)	−0.449 to 0.279	−1.004	.279	0.000	0.000
Sleep and relaxation	3	4	0.081 (0.126)	−0.320 to 0.482	0.641	.567	0.000	0.018

ES = effect size (Hedges *g*); SE = standard error; CI = confidence interval; CVD = cardiovascular disease; PA = physical activity.

* *p* < .05.

** *p* < .01.

*** *p* < .001.

We conducted additional analyses for each outcome category separately. For the clinical outcome measures, we found significant mean effect sizes of eHealth lifestyle interventions on glucose outcomes (0.16, *p* = .022), weight outcomes (0.12, *p* = .015), and physical activity capacity outcomes (0.14, *p* < .001), but not for eHealth lifestyle interventions on blood pressure outcomes, cholesterol outcomes, and composite score outcomes. For the behavioral outcome measures, we found significant mean effect sizes of eHealth lifestyle interventions and physical activity outcomes (0.17, *p* < .001) and nutrition outcomes (0.13, *p* = .007), but not for eHealth lifestyle interventions on smoking outcomes, alcohol outcomes, and sleep and relaxation outcomes. See Table 1 for all mean effect sizes of eHealth lifestyle interventions on each outcome category.

### Heterogeneity

Given the three-level model, we assessed both between-study heterogeneity (variance between studies) and within-study heterogeneity (variance between effect sizes from the same study). For all outcomes, we found significant between-study heterogeneity (*σ*^2^ = 0.014, *χ*^2^(1) = 29.53, *p* < .001) and within-study heterogeneity (*σ*^2^ = 0.055, *χ*^2^(1) = 499.77, *p* < .001). For clinical outcomes, the between-study heterogeneity (*σ*^2^ = 0.010, *χ*^2^(1) = 8.92, *p* = .003) and within-study heterogeneity (*σ*^2^ = 0.064, *χ*^2^(1) = 440.83, *p* < .001) were also significant. Also, for behavioral outcomes, we found a significant between-study heterogeneity (*σ*^2^ = 0.034, *χ*^2^(1) = 22.83, *p* < .001) and within-study heterogeneity (*σ*^2^ = 0.021, *χ*^2^(1) = 26.07, *p* < .001). Given these significant heterogeneity values, we conducted moderator analyses for all outcomes combined, for clinical outcomes, and for behavioral outcomes separately (Table 1).

### Moderator Analyses

#### Intervention Type, Delivery Mode, and Dose of Support

To test the effect of intervention type (self-help versus human-supported), dose of human support (minor versus major), and delivery mode of human support (remote versus blended) on the relationship between eHealth lifestyle interventions and clinical and behavioral health outcomes, we conducted moderator analyses. We found that intervention type did not moderate the mean effect size of eHealth lifestyle interventions on all health outcomes (clinical and behavioral health outcomes combined; *p* = .169; Table 2). Moreover, both dose (*p* = .698) and delivery mode of human support (*p* = .557) did not moderate the mean effect size eHealth lifestyle interventions on all health outcomes (clinical and behavioral health outcomes combined). We performed the same moderator analyses on the mean effect size of eHealth lifestyle interventions and on both clinical and behavioral outcomes separately (Table 2). For clinical outcomes, we again found no significant moderator effect of intervention type (*p* = .374), dose of human support (*p* = .439), or delivery mode (*p* = .308). For behavioral outcomes, we also found no significant moderator effect of intervention type (*p* = .080), dose of human support (*p* = .272), or delivery mode (*p* = .144).

**TABLE 2:** Results for the Moderator Analyses of Intervention Type, Dose of Human Support, and Delivery Mode of Human Support on the Association Between eHealth Interventions and Clinical and Behavioral Health Outcomes

Moderator	No. Studies	No. ES	Overall Test	*p* of Overall Test	Mean ES (SE)	95% CI	*t* Value	*p* of ES
All outcomes								
Intervention type	102	809	*F*(1,807) = 1.900	.169				
Self-help interventions					0.137 (0.032)	0.074 to 0.201	4.241	<.001***
Human-supported interventions					0.086 (0.020)	0.047 to 0.125	4.292	<.001***
Dose of human support	76	590	*F*(1,588) = .150	0.698				
Minor level					0.105 (0.036)	0.034 to 0.176	2.907	.004**
Major level					0.087 (0.031)	0.027 to 0.147	2.839	.005**
Delivery mode of human support	75	586	*F*(1,584) = .346	.557				
Remote					0.102 (0.026)	0.052 to 0.152	3.988	<.001***
Blended					0.080 (0.036)	0.010 to 0.150	2.250	.025*
Clinical outcomes								
Intervention type	92	597	*F*(1,595) = .792	.374				
Self-help interventions					.113 (0.035)	0.044 to 0.182	3.204	.001**
Human-supported interventions					.077 (0.021)	0.035 to 0.118	3.610	<.001***
Dose of human support	69	440	*F*(1,438) = .599	.439				
Minor level					.111 (0.041)	0.030 to 0.191	2.696	.007**
Major level					.068 (0.037)	−0.005 to 0.142	1.834	.067^†^
Delivery mode of human support	68	436	*F*(1,434) = 1.041	.308				
Remote					0.099 (0.029)	0.042 to 0.157	3.386	<.001***
Blended					0.063 (0.038)	−0.012 to 0.137	1.653	.099^†^
Behavioral outcomes								
Intervention type	60	212	*F*(1,210) = 3.100	.080^†^				
Self-help interventions					0.207 (0.053)	0.102 to 0.312	3.886	<.001***
Human-supported interventions					0.101 (0.034)	0.034 to 0.167	2.993	.003**
Dose of human support	44	150	*F*(1,148) = 1.215	.272				
Minor level					0.038 (0.058)	−0.076 to 0.153	0.662	.509
Major level					0.117 (0.042)	0.034 to 0.201	2.790	.006**
Delivery mode of human support	44	150	*F*(1,148) = 2.159	.144				
Remote					0.062 (0.038)	−0.014 to 0.138	1.614	.109
Blended					0.167 (0.062)	0.044 to 0.290	2.679	.008**

ES = effect size (Hedges *g*); SE = standard error; CI = confidence interval.

* *p* < .05.

** *p* < .01.

*** *p* < .001.

^†^ *p* < .10.

#### Study, Intervention and Population Characteristics

We conducted several additional moderator analyses to explore whether study, intervention, or population characteristics could explain this heterogeneity (Table 3). Control condition type (passive versus active; *p* = .344), intervention length (*p* = .588), mean sample age (*p* = .053), or diagnosis (CVD, T1DM, T2DM, CKD, at-risk, or mixed; *p* = .197) did not significantly moderate the mean effect size of eHealth lifestyle interventions on all health outcomes, or on either clinical or behavioral health outcomes separately (Table 3).

**TABLE 3:** Results for the Moderator Analyses of Study, Intervention, and Population Characteristics on the Association Between eHealth Interventions and Clinical and Behavioral Health Outcomes

Moderator	No. Studies	No. ES	Overall Test	*p* of Overall Test	Mean ES (SE)*^a^*	95% CI	*t* Value	*p* of ES
All outcomes								
Outcome type	102	809	*F*(1,807) = 3.810	.051^†^				
Clinical outcomes					0.086 (0.019)	0.049 to 0.124	4.500	<.001***
Behavioral outcomes					0.142 (0.028)	0.087 to 0.196	5.109	<.001***
Control condition type	102	809	*F*(1,807) = .897	.344				
Passive					0.110 (0.021)	0.070 to 0.151	5.320	<.001***
Active					0.071 (0.036)	−0.000 to 0.142	1.959	.050^†^
Intervention length	101	805	*F*(1,803) = .294	.588	0.117 (0.031)	0.056 to 0.177	3.792	<.001***
Mean sample age	91	750	*F*(1,748) = 3.758	.053^†^	0.198 (0.053)	0.095 to 0.301	3.760	<.001***
Diagnosis	102	809	*F*(5,803) = 1.470	.197				
CVD					0.146 (0.037)	0.075 to 0.218	4.000	<.001***
T1DM					0.034 (0.212)	−0.382 to 0.450	0.161	.872
T2DM					0.104 (0.030)	0.046 to 0.162	3.497	<.001***
CKD					0.024 (0.098)	−0.168 to 0.215	0.242	.809
At-risk					0.126 (0.038)	0.051 to 0.202	3.286	.001**
Mixed patient group					0.002 (0.046)	−0.089 to 0.092	0.040	.968
Clinical outcomes								
Control condition type	92	597	*F*(1,595) = .653	.420				
Passive					0.095 (0.021)	0.053 to 0.137	4.451	<.001***
Active					0.059 (0.039)	−0.016 to 0.135	1.538	.125
Intervention length	91	596	*F*(1,594) = .025	.873	0.094 (0.033)	0.029 to 0.158	2.864	.004**
Mean sample age	83	551	*F*(1,549) = 2.750	.098	0.179 (0.058)	0.065 to 0.292	3.095	.002**
Diagnosis	92	597	*F*(5,591) = 1.244	.287				
CVD					0.135 (0.039)	0.059 to 0.211	3.490	<.001***
T1DM					0.183 (0.378)	−0.560 to 0.926	0.483	.629
T2DM					0.095 (0.031)	0.034 to 0.156	3.062	.002**
CKD					0.028 (0.096)	−0.161 to 0.218	0.295	.768
At-risk					0.095 (0.040)	0.017 to 0.173	2.390	.017*
Mixed patient group					−0.015 (0.050)	−0.114 to 0.083	−0.015	.762
Behavioral outcomes								
Control condition type	60	212	*F*(1,210) = .211	.646				
Passive					0.141 (0.038)	0.066 to 0.217	3.692	<.001***
Active					0.109 (0.058)	−0.004 to 0.223	1.900	.059^†^
Intervention length	59	209	*F*(1,207) = 1.242	.266	0.188 (0.057)	0.076 to 0.301	3.298	<.001**
Mean sample age	55	199	*F*(1,197) = 2.441	.120	0.243 (0.082)	0.081 to 0.405	2.960	.003**
Diagnosis	60	212	*F*(5,206) = .508	.770				
CVD					0.122 (0.062)	−0.001 to 0.245	1.955	.052^†^
T1DM					−0.016 (0.253)	−0.514 to 0.482	−0.063	.950
T2DM					0.155 (0.060)	0.037 to 0.273	2.595	.010*
CKD					−0.003 (0.212)	−0.420 to 0.415	−0.013	.990
At-risk					0.196 (0.070)	0.058 to 0.334	2.803	.006**
Mixed patient group					0.064 (0.076)	−0.086 to 0.215	0.843	.400

ES = effect size (Hedges *g*); SE = standard error; CI = confidence interval; CVD = cardiovascular disease; T1DM = type 1 diabetes mellitus; T2DM = type 2 diabetes mellitus; CKD = chronic kidney disease.

* *p* < .05.

** *p* < .01.

*** *p* < .001.

^†^ *p* < .10.

*^a^* Continuous predictors represent the ES size of a participant with an average value on the corresponding predictor.

## DISCUSSION

Our multilevel meta-analysis demonstrated that eHealth interventions are effective in improving cardiometabolic health outcomes. However, overall effect size, both on clinical and behavioral health outcomes, was small. The small effect sizes are comparable to other meta-analyses investigating eHealth lifestyle interventions (e.g., 0.139 in Ref. (17); 0.16 in Ref. (19)). More specifically, eHealth lifestyle interventions positively influenced the clinical health outcomes glucose, weight, and physical activity capacity (but not blood pressure, cholesterol, and CVD composite score) and the following behavioral health outcomes: physical activity behavior, and nutrition (but not smoking, alcohol, and sleep or relaxation). Furthermore, we found that study, intervention, or sample characteristics did not impact the positive effect of eHealth lifestyle interventions on health outcomes. Finally, control group type, intervention length, mean sample age, and diagnosis did not influence the effect of eHealth lifestyle interventions on clinical and behavioral health outcomes.

Contrary to our expectations, our meta-analysis did not show the expected difference between human-supported and self-help eHealth interventions. Both human-supported and self-help eHealth interventions were effective in improving clinical and behavioral health outcomes. Our results contrast other meta-analyses (14–16) that did find a stronger effect of human support in eHealth interventions on improving cardiometabolic risk factors, or a more pronounced effect of blended interventions compared with remotely supported ones. Instead, our results are more in line with studies that indicated that there is no difference in the improvement of cardiometabolic risk factors between human-supported and self-help eHealth interventions (17,19), or blended and remotely supported eHealth interventions (18). Although one of the aims of this meta-analysis was to find an explanation for the inconsistent results of human support in eHealth interventions in these different meta-analyses, our results with regard to dose and delivery mode of the support did not provide this explanation. However, these inconsistencies could be due to population-, outcome-, or intervention-related factors.

Regarding the first factor that could provide an explanation to inconsistent results of human support, the study population, meta-analyses focusing on the general population did not find a difference between human-supported and self-help eHealth interventions (17,19). However, contrary to our results, those studies that focused on a patient or at-risk population did encounter differences between human-supported and self-help eHealth interventions (14–16). Our meta-analysis with patients and an at-risk population did not find these differences, and also no differences between conditions. We did, however, find that age had a borderline significant effect. Possibly, patients are generally older and therefore more in need of human support when using eHealth compared with the general population (36).

With regard to outcome-related factors, our study showed no difference between human-supported and self-help eHealth interventions in the outcome measure we used: effectiveness. Possibly, we would have found a difference if we used intervention adherence as an outcome measure. Multiple studies have shown that self-help eHealth interventions suffer from low levels of intervention adherence, which refers to the extent to which the individual uses the intervention as intended (37–40). However, meta-analyses on multiple studies investigating intervention adherence to eHealth interventions are difficult to conduct because only a small proportion of the studies report eHealth intervention adherence (41). Because intervention adherence is related to intervention effectiveness (42), the level of intervention adherence could possibly be the missing explanation for the inconsistent results found in previous meta-analyses regarding the possible added contribution of human support to self-help eHealth interventions.

Finally, the effectiveness of human support in eHealth interventions could depend on the characteristics of the specific interventions. In our study, the inclusion criteria were that the tested intervention was delivered via a website or mobile-based application, provided education or skills training, and was interactive. This narrowed down the type of interventions included in our analyses, which may have positively influenced the quality of both human-supported and self-help interventions, and consequently reduced the difference in effectiveness between the two. Some meta-analyses did find a lower effectiveness of self-help eHealth interventions, possibly because they included a broader variety of interventions, including interventions lacking important behavior change techniques or lower quality of interactive components. For example, Beishuizen and colleagues (16) included interventions without education or skills training, Lau and colleagues (14) included interventions that were not interactive, and Joiner and colleagues (15) included any intervention that used some form of digital communication (including social media, DVDs, or videoconferencing). We know that interventions that are more elaborate, for example, because they incorporate multiple behavior change techniques, are more effective in improving health behavior (19). It is therefore not surprising that automated support is frequently combined with behavior change techniques and persuasive system design principles (43). Furthermore, an advantage of self-help interventions is that users can customize what behavior change technique features are used in their eHealth intervention, which users appreciate (13). This means that cardiometabolic patients themselves can decide whether their eHealth lifestyle intervention shows motivational messages (e.g., through push messages on their smartphone) or not, in what frequency they want to track their behaviors (e.g., filling in a food diary daily or weekly), or whether they want to watch all the educational videos or whether they already have enough knowledge on the topic. Therefore, the more thorough implementation of behavior change techniques and interactive components, as well as the freedom for the user to choose, could positively affect the quality of self-help eHealth interventions.

Finally, it is important to note that our hypotheses with regard to the dose and delivery mode of support were based on findings in mental health interventions. Meta-analyses focusing on both eHealth and regular interventions or eHealth interventions only, aimed at patients with obsessive-compulsive disorder (20), depression and anxiety (22,44,45), or mental disorders in general (21), found that human-supported interventions are more effective and that higher levels of support lead to higher effect sizes. Our meta-analysis, however, focused on lifestyle interventions showed contradictory results. Possibly, mental health issues require more complex interventions that might require more human support than self-help interventions. Future studies could investigate whether interventions aimed at mental health improvement necessitate (more extensive) human support compared with interventions aimed at lifestyle behavior change.

### Strengths, Limitations, and Future Research

A strength of our study is that we used a precise definition of eHealth as a study eligibility criterion. As noted previously, many other meta-analyses on eHealth included a larger variety of digital tools (e.g., DVDs and videoconferencing) or less elaborate types of eHealth interventions (e.g., without interactive or educational components). Our definition created more homogeneity in the inclusion of eHealth studies. Another strength would be the inclusion of four different types of cardiometabolic diseases. Not only is there a high comorbidity between CVD, CKD, T1DM, and T2DM, but they also share similar underlying risk factors and have a similar management strategy (i.e., lifestyle modifications; (4,5)). Finally, another advantage of our study was our multilevel approach for the meta-analysis. Other studies concerning eHealth lifestyle interventions for people with cardiometabolic diseases used a more traditional univariate approach and conducted a meta-analysis for each outcome separately. We contributed to these studies by applying a three-level model approach (30), which does not only deal with interdependency of effect sizes but also present an overall picture of the effect of eHealth lifestyle interventions on clinical and behavioral health outcomes.

A number of limitations need to be considered. First, our sensitivity analyses revealed that there was some publication bias. This may have caused the mean effect sizes in our study to be different from the true effect sizes for the effect of eHealth interventions on clinical and behavioral cardiometabolic health outcomes. The results should therefore be interpreted with caution. Another limitation of the study was the methodological quality of the included studies. The risk of bias assessment resulted in a generally good evaluations of the studies, and we found that the risk of bias assessment had no moderating effect on the relation between eHealth intervention and clinical and behavioral health outcomes. Because self-management interventions cannot be blinded, almost all studies lacked double blinding, leading to a possible risk of bias due to deviations from the intended interventions and in measurement of the outcome (e.g., health professionals measuring participants blood pressure or weight). Furthermore, only a minority of the studies preregistered their study and analyses, which may cause a risk of bias in selection of the reported results. As another limitation, we should mention that the included studies were substantially heterogeneous on several levels. With regard to the control group, some of the studies had a passive control group (waitlist or care as usual), whereas in other studies, patients in the control group received another intervention. Furthermore, there was a large variety in intervention duration, which ranged from 1.5 to 24 months. There were also big differences in mean age of the study samples, which varied from 35.2 to 75.9 years. Despite this, our analyses revealed that control group type, intervention length, and mean sample age had no moderating effect on the relation between eHealth intervention and clinical and behavioral health outcomes.

Our study has raised new questions regarding eHealth interventions and human support that would be interesting to address in future research. Adherence to interventions is still poorly defined and underreported. Therefore, we suggest that future randomized controlled trials evaluating eHealth lifestyle interventions implement better intervention adherence measures. This would also enable the investigation of the relationship between human-supported and self-help eHealth interventions on intervention adherence. In contrast to previous meta-analyses, our study did not find a difference between these human-supported and self-help eHealth interventions. As stated before, this inconsistency could be due to the quality of eHealth interventions, as we had strict inclusion and exclusion criteria with regard to the way eHealth interventions were designed and executed. Future meta-analyses could investigate what components make self-help eHealth interventions as effective as human-supported eHealth interventions, and whether lower-quality eHealth interventions benefit more from applying human support. Another suggestion for future research would be to further investigate the need for human support for specific subgroups of patients. For example, it is important to specifically examine implementation, intervention adherence, and effects of eHealth interventions for patients of lower socioeconomic status including those with less digital literacy or resources (46). These variables were reported inconsistently among the included studies and require more attention in future studies. Moreover, meta-analyses on interventions for psychological outcomes instead of lifestyle outcomes indicate that human support is particularly important for cognitive-behavioral interventions focusing on psychological distress and related outcomes (20–22,44,45). Therefore, it would be interesting to investigate whether mental health also influences the need for support in eHealth lifestyle interventions. Finally, the studies included in our analyses were heterogenous in regard to patient groups and outcomes. Most studies focused on CVD and T2DM patients and, to a lesser degree, on patients with T1DM and CKD. Despite alcohol use and sleep and relaxation being important risk factors to be address in cardiometabolic disease management, only very few studies targeted these health outcomes. It would therefore be important for eHealth researchers to also focus on these less represented patient groups and behavioral risk factors in future studies.

### Conclusion

Our meta-analysis demonstrated that eHealth lifestyle interventions are effective in improving clinical and behavioral health outcomes among people with cardiometabolic diseases. However, there was no difference between self-help and human-supported eHealth interventions’ effectiveness. Neither dose nor delivery mode of support affected human-supported intervention effectiveness. Several population-, outcome-, and intervention-related factors were ruled out as possible moderators of these relationships. These findings add substantially to our understanding of the role of human support in lifestyle eHealth interventions, which is important to make lifestyle interventions accessible for a larger and more varied audience. Although further research is required to unravel the possible added contribution of human support for specific eHealth interventions in subgroups of patients, our results seem promising for the broad application of self-help eHealth interventions in cardiometabolic diseases.

## Supplementary Material
