# Supplementary material for: Effectiveness of Human-Supported and Self-Help eHealth Lifestyle Interventions for Patients With Cardiometabolic Risk Factors: A Meta-Analysis
Source: Psychosom Med. 2023 Oct 9;85(9):795–804. doi: 10.1097/PSY.0000000000001242 (PMC10662612; doi:10.1097/PSY.0000000000001242)
Supplement: Supplementary file 1 [file psymed-85-795-s001.docx]

**Supplemental Digital Content**

**Search string.**

Randomized controlled trials about eHealth lifestyle interventions to promote lifestyle modification in cardiometabolic disease patients.

(("app"[tiab] OR "apps"[tiab] OR "digital"[ti] OR "e health"[tiab] OR "econsult*"[tiab] OR "e-consult*"[tiab] OR "ehealth"[tiab] OR "e-health"[tiab] OR "electronic communication*"[tiab] OR "Electronic Learning"[tiab] OR "iCBT"[tiab] OR "internet"[ti] OR "m health"[tiab] OR "mhealth"[tiab] OR "m-health"[tiab] OR "mobile app"[tiab] OR "mobile application"[tiab] OR "mobile application"[tiab] OR "mobile applications"[tiab] OR "mobile apps"[tiab] OR "mobile health"[tiab] OR "mobile"[ti] OR "on line"[ti] OR "online therapy"[tiab] OR "online"[ti] OR "on-line"[ti] OR "personal digital assistant"[tiab] OR "remote communication"[tiab] OR "remote computer"[tiab] OR "remote computers"[tiab] OR "remote consultation"[tiab] OR "remote health care"[tiab] OR "remote healthcare"[tiab] OR "remote monitoring"[tiab] OR "remote system"[tiab] OR "remote systems"[tiab] OR "remote technologies"[tiab] OR "remote technology"[tiab] OR "remote"[tiab] OR "smart technol*"[tiab] OR "smart technology"[tiab] OR "tele health"[tiab] OR "telecare"[tiab] OR "tele-care"[tiab] OR "teleconsult*"[tiab] OR "teleconsultation"[tiab] OR "telehealth"[tiab] OR "tele-health"[tiab] OR "telemed*"[tiab] OR "Telemedicine"[majr] OR "telemedicine"[tiab] OR "telemonitoring"[tiab] OR "tele-monitoring"[tiab] OR "telenurs*"[tiab] OR "telenursing"[tiab] OR "telerehabilitation"[tiab] OR "wearable technologies"[tiab] OR "wearable technology"[tiab] OR "web access"[tiab] OR "web application"[tiab] OR "web applications"[tiab] OR "web based"[tiab] OR "web portal*"[ti] OR "web"[ti] OR "webapp*"[tiab] OR "webbased"[tiab] OR "web-based"[tiab] OR "webpage"[tiab] OR "webpages"[tiab] OR "website"[ti] OR "websites"[tiab] OR "mobile application"[tiab] OR "computer application"[tiab] OR "internet"[tiab] OR "telecommunications"[tiab] OR "mobile applications"[tiab] OR "computer applications"[tiab] OR "internet"[majr] OR "telecommunications"[majr]) AND ("Life style"[majr] OR "life style"[tiab] OR "lifestyle"[tiab] OR "life-style"[tiab] OR "life styles"[tiab] OR "lifestyles"[tiab] OR "life-styles"[tiab] OR "Health behavior"[majr] OR "health behaviour"[tiab] OR "health behavior"[tiab] OR "Health promotion"[majr] OR "health promotion"[tiab] OR "self-management"[tiab] OR "self management"[tiab] OR "Exercise"[majr] OR "exercise"[tiab] OR "physical activity"[tiab] OR "Diet"[majr] OR "Diet Therapy"[majr] OR "diet"[tiab] OR "diets"[tiab] OR "dietary"[tiab] OR "fat"[tiab] OR "salt"[tiab] OR "natrium"[tiab] OR "sodium"[tiab] OR "Dietary Carbohydrates"[majr] OR carbohydrate*[tiab] OR "calories"[tiab] OR "Dietary Proteins"[majr] OR "proteins"[tiab] OR "fat intake"[tiab] OR "salt intake"[tiab] OR "Eating"[majr] OR "eating"[tiab] OR "Nutrition Therapy"[majr] OR "nutrition"[tiab] OR "Smoking"[majr] OR "smoking"[tiab] OR "Tobacco use"[majr] OR "tobacco"[tiab] OR "nicotine"[tiab] OR "cigarettes"[tiab] OR "cigarette"[tiab] OR "Drinking Behavior"[majr] OR "alcohol drinking"[majr] OR "Alcohol Abstinence"[tiab] OR "Alcohol Drinking"[tiab] OR "alcohol"[tiab] OR "drinking"[majr] OR "drinking"[tiab] OR "Sleep"[majr] OR "sleep"[tiab] OR "sedentary"[tiab] OR "Medication Adherence"[majr] OR "Medication Adherence"[tiab] OR "medication"[tiab] OR "Body Weight"[majr] OR "weight"[tiab] OR "body weight"[tiab] OR "Blood glucose"[majr] OR "blood glucose"[tiab] OR "BMI"[tiab] OR "Body mass index"[majr] OR "body mass index"[tiab] OR "overweight"[majr] OR "overweight"[tiab] OR "obesity"[majr] OR "obesity"[tiab] OR "obese"[tiab] OR "Blood pressure"[majr] OR "Hypertension"[majr] OR "blood pressure"[tiab] OR "blood pressure determination"[majr] OR "Cholesterol"[majr] OR "cholesterol"[tiab] OR "triglycerides"[majr] OR "triglycerides"[tiab] OR "triglyceride"[tiab] OR "glycated hemoglobin A"[tiab] OR "hemoglobin A1c"[tiab] OR "HbA1c"[tiab] OR "glomerular filtration rate"[tiab] OR "GFR"[tiab] OR "glucoregulation"[tiab] OR "cardiac rehabilitation"[tiab]) AND ("Cardiovascular Diseases"[majr] OR "cardiovascular disease"[tiab] OR "cardiovascular diseases"[tiab] OR "CVD"[tiab] OR "cardiac"[tiab] OR "heart disease"[tiab] OR "coronary heart disease"[tiab] OR "coronary artery disease"[tiab] OR "myocardial infarction"[tiab] OR "myocard infarct"[tiab] OR "myocard infarction"[tiab] OR "myocardial infarct"[tiab] OR "myocardial ischemia"[tiab] OR "angina pectoris"[tiab] OR "angina"[tiab] OR "heart infarct"[tiab] OR "heart infarction"[tiab] OR "heart attack"[tiab] OR "heart failure"[tiab] OR "cardiomyopathy"[tiab] OR **"Stroke"[majr] OR** "stroke"[tiab] OR "cerebrovascular accident"[tiab] OR "transient ischaemic attack"[tiab] OR "transient ischemic attack"[tiab] OR "TIA"[tiab] **OR "cerebrovascular accidents"[tiab] OR "transient ischaemic attacks"[tiab] OR "transient ischemic attacks"[tiab] OR "TIAs"[tiab]** OR "percutaneous angioplasty"[tiab] OR "PTCA"[tiab] OR "PCI"[tiab] OR "coronary bypass surgery"[tiab] OR "coronary artery bypass graft"[tiab] OR "aortic aneurysm"[majr] OR "aortic aneurysm"[tiab] OR "peripheral artery disease"[tiab] OR "peripheral arterial disease"[tiab] OR "peripheral vascular disease"[tiab] OR "Reversible Ischemic Neurological Deficit"[tiab] OR "RIND"[tiab] OR Cardiometabolic*[tiab] OR "Renal Insufficiency, Chronic"[majr] OR "renal insufficiency"[tiab] OR "kidney insufficiency"[tiab] OR "kidney disease"[tiab] OR "renal disease"[tiab] **OR "kidney diseases"[tiab] OR "renal diseases"[tiab]** OR "kidney failure"[tiab] OR "renal failure"[tiab] OR "chronic kidney"[tiab] OR "chronic renal"[tiab] OR "end stage kidney"[tiab] OR "end-stage kidney"[tiab] OR "end stage renal"[tiab] OR "end-stage renal"[tiab] OR "advanced kidney"[tiab] OR "advanced renal"[tiab] **OR "ESRD"[tiab]** OR "Renal Replacement Therapy"[majr] OR "Renal Dialysis"[majr] OR "renal dialysis"[tiab] OR "Peritoneal Dialysis"[majr] OR "peritoneal dialysis"[tiab] OR "pre-dialysis"[tiab] OR "predialysis"[tiab] OR "dialysis"[tiab] OR "hemodialysis"[tiab] OR "haemodialysis"[tiab] OR "hemodiafiltration"[tiab] **OR "Kidney Transplantation"[majr]** OR kidney transplant*[tiab] OR renal transplant*[tiab] OR "RRT"[tiab] OR "CKD"[tiab] OR "CKF"[tiab] OR "CRD"[tiab] OR "CRF"[tiab] OR "ESKD"[tiab] OR "ESRD"[tiab] OR "ESKF"[tiab] OR "ESRF"[tiab] OR "Diabetes Mellitus, Type 2"[majr:noexp] OR type 2 diab*[tiab] OR type II diab*[tiab] OR non-insulin-dependent diab*[tiab] OR adult-onset diab*[tiab] OR "T2DM"[tiab] OR "T2D"[tiab] OR "TIIDM"[tiab] OR "TIID"[tiab] OR "DM2"[tiab] OR "Diabetes Mellitus"[majr] OR "diabetes mellitus"[tiab] OR "Diabetes Mellitus, Type 1"[majr:noexp] OR type 1 diab*[tiab] OR type I diab*[tiab] OR insulin-dependent diab*[tiab] OR juvenile diab*[tiab] OR juvenile-onset diab*[tiab] OR juvenile onset diab*[tiab] OR "IDDM"[tiab] OR "T1DM"[tiab] OR "TIDM"[tiab] OR autoimmune diab*[tiab] OR "DM1"[tiab]) AND ("Randomized Controlled Trial"[Publication Type] OR "randomized"[ti] OR "randomised"[ti] OR "RCT"[ti] OR random*[ti] OR "single blind"[ti] OR "double blind"[ti] OR "triple blind"[ti] OR single blind*[ti] OR double blind*[ti] OR triple blind*[ti] OR "Equivalence Trial"[ti] OR "Pragmatic Clinical Trial"[ti] OR "Randomized Controlled Trials as Topic"[Mesh] OR "Equivalence Trial"[Publication Type] OR "Equivalence Trials as Topic"[Mesh] OR "superiority trial"[ti] OR "non-inferiority trial"[ti]) AND (english[la] OR dutch[la]) AND ("1995/01/01"[PDAT] : "3000/12/31"[PDAT]))

**Table S1.** Characteristics of the included studies.

| **Study** | **Participants** | **Age (mean)** | **Gender (% female)** | **RCT setting** | **Intervention group(s)** | **Support type** | **Control group** | **Outcomes types** |
| --- | --- | --- | --- | --- | --- | --- | --- | --- |
| Agarwal 2019(a) | 50 T2DM and hypertension patients | 64.1 | 44.5 | 2-arm; Canada | 4-month mobile-based intervention aimed at physical activity, nutrition, and sleep | Human-supported, blended, major, layperson | Usual care | Glucose, physical activity behavior |
| Agarwal 2019(b) | 223 T2DM patients | 51.8 | 47.5 | 2-arm; Canada | 3-month mobile-based intervention, aimed at physical activity and nutrition | Self-help | Waitlist | Glucose |
| Akinci 2019 | 66 T2DM patients | 52.5 | 72.3 | 3-arm*; Finland | 2-month web-based intervention aimed at physical activity | Human-supported, remote only, minor, layperson | Usual care | Glucose, cholesterol, weight, physical activity capacity, physical activity behavior |
| Appel 2011 | 415 CVD patients | 54.0 | 63.6 | 3-arm; USA | 24-month web-based interventions aimed at physical activity and nutrition | (1) Human-supported, remote, minor, professional  (2) Human-supported, blended, major, professional | Usual care | Weight |
| Baer 2020 | 840 T2DM patients | 59.3 | 60.0 | 3-arm; USA | 12-month web-based interventions aimed at physical activity and nutrition | (1) Self-help  (2) Human-supported, remote, minor, professional | Usual care | Weight |
| Bailey 2020 | 20 T2DM patients | 56.0 | 45.0 | 2-arm; UK | 2-month mobile-based intervention aimed at physical activity | Self-help | Usual care | Blood pressure, glucose, weight, physical activity capacity, physical activity behavior |
| Bennett 2010 | 101 CVD patients | 54.4 | 47.5 | 2-arm; USA | 3-month web-based intervention aimed at physical activity and nutrition | Human-supported, blended, minor, professional | Usual care | Blood pressure, weight |
| Bennett 2018 | 351 hypertension, diabetes, hyperlipidemia, and obesity patients | 50.7 | 68.0 | 2-arm; USA | 12-month mobile-based intervention aimed at nutrition | Human-supported, blended, minor, professional | Usual care | Blood pressure, glucose, cholesterol, weight |
| Bond2007 | 62 T1DM and T2DM patients | 67.2 | 45.0 | 2-arm; USA | 6-month web-based intervention aimed at nutrition and physical activity | Human-supported, remote, major, professional | Usual care | Blood pressure, glucose, cholesterol, weight |
| Bozorgi 2021 | 120 patients with hypertension | 51.8 | 41.0 | 2-arm; Iran | 2-month mobile-based intervention aimed at nutrition and smoking | Self-help | Usual care | Blood pressure, weight, physical activity capacity, physical activity behavior, nutrition |
| Cai 2021 | 100 CVD patients | 57.0 | 35.0 | 2-arm; China | 3-month mobile-based intervention aimed at physical activity | Human-supported, remote, minor, professional | Usual care | Physical activity capacity, physical activity behavior |
| Chao2019 | 121 T2DM patients | N.A. | 39.0 | 2-arm; Taiwan | 18-month mobile-based intervention aimed at physical activity and nutrition | Human-supported, remote, minor, professional | Usual care | Blood pressure, glucose, weight |
| Choi2019 | 100 CVD patients | 56.9 | 38.9 | 2-arm; USA | 3-month mobile-based intervention aimed at nutrition | Human-supported, blended, major, professional | Usual care | Blood pressure, glucose, cholesterol, weight |
| Chow2021 | 41 at-risk population | N.A | 49.4 | 2-arm; USA | 4-month mobile-based intervention aimed at smoking, physical activity and nutrition | Human-supported, remote, major, professional | Other eHealth intervention | Physical activity behavior, nutrition |
| Connely2017 | 31 T2DM patients | 66.7 | 41.7 | 3-arm; UK | 6-month web-based intervention aimed at physical activity | (1) Self-help  (2) Human-supported, blended, minor, professional | Non eHealth intervention | Weight, glucose, physical activity behavior |
| Dorje2019 | 312 CVD patients | 60.5 | 18.5 | 2-arm; China | 6-month mobile-based intervention aimed at physical activity and nutrition | Human-supported, remote, minor, professional | Usual care | Blood pressure , cholesterol, weight |
| Dorsch2020 | 50 CVD patients | 57.4 | 59.5 | 2-arm; USA | 2-month mobile-based intervention aimed at nutrition | Self-help | Usual care | Blood pressure, nutrition |
| Duan2018 | 114 CVD patients | 48.7 | 53.2 | 2-arm; USA | 2-month mobile-based intervention aimed at physical activity and nutrition | Human-supported, remote, minor, professional | Waitlist | Physical activity behavior, nutrition |
| Duscha2018 | 32 CVD patients | 63.2 | 52.1 | 2-arm; China | 3-month web-based intervention aimed at physical activity | Human-supported, remote, major, professional | Usual care | Physical activity capacity, physical activity behavior |
| Engelen2020 | 208 CVD patients | 63.5 | 31.5 | 2-arm; The Netherlands | 12-month Web-based intervention aimed at smoking, alcohol, and physical activity | Self-help | Usual care | Blood pressure, cholesterol, weight, physical activity behavior, nutrition, alcohol |
| Fukuoka2015 | 61 at-risk population | 55.3 | 77.1 | 2-arm; USA | 5-month mobile-based intervention aimed at physical activity and nutrition | Human-supported, blended, major, layperson | Usual care | Blood pressure, glucose, cholesterol, weight |
| Glasgow2012 | 463 T2DM patients | 58.7 | 77.4 | 3-arm; USA | 4-month web-based intervention aimed at physical activity and nutrition | 1. Self-help 2. Human supported, remote, major, professional | Other eHealth intervention | Blood pressure, glucose, cholesterol, weight, cardiovascular compost score, physical activity behavior, nutrition |
| Gong2020 | 187 T2DM patients | 56.9 | 41.8 | 2-arm; Australia | 12-month mobile-based intervention aimed at physical activity and nutrition | Human-supported, remote, minor, layperson | Waitlist | Glucose, weight |
| Grau-Pellicer2020 | 41 CVD patients | 65.8 | 49.4 | 2-arm; Spain | 3-month mobile-based intervention aimed at physical activity | Human-supported, blended, major, professional | Usual care | Physical activity behavior |
| Gunawardena2019 | 67 T1DM and T2DM patients | 52.5 | 40.0 | 2-arm; Sri Lanka | 6-month mobile-based intervention aimed at nutrition and physical activity | Self-help | Usual care | Glucose |
| Hansel2017 | 120 T2DM patients | 56.6 | 66.7 | 2-arm; France | 4-month web-based intervention aimed at nutrition and physical activity | Self-help | Usual care | Glucose, cholesterol, weight, nutrition |
| Haste2017 | 61 T2DM patients | N.A. | N.A. | 2-arm; UK | 12-month web-based intervention aimed at nutrition and physical activity | Human-supported, remote, minor, professional | Usual care | Weight |
| Hilmarsdóttir2021 | 37 T2DM patients | 51.2 | 63.4 | 2-arm; Iceland | 6-month mobile-based intervention aimed at nutrition, stress and physical activity | Human-supported, remote, minor, layperson | Waitlist | Blood pressure, glucose, cholesterol, weight |
| Höchsmann2019 | 36 T2DM patients | N.A. | 52.8 | 2-arm; Switzerland | 6-month mobile-based intervention aimed at physical activity | Self-help | Non eHealth intervention | Blood pressure, glucose, cholesterol, weight, physical activity behavior |
| Holmen2014 | 151 T2DM patients | 57.3 | 36.5 | 3-arm; Norway | 12-month mobile-based intervention aimed at nutrition and physical activity | Self-help | Usual care | Glucose, weight |
| Houchen-Wolloff2018 | 60 CVD patients | 61.5 | 10.0 | 2-arm; UK | 2-month web-based intervention aimed at physical activity, nutrition, stress and smoking | Human-supported, remote, minor, professional | Usual care | Physical activity capacity |
| Humalda2020 | 99 CKD patients | 56.7 | 16.0 | 2-arm; The Netherlands | 9-month web-based intervention aimed at nutrition | Human-supported, blended, major, professional | Usual care | Blood pressure, nutrition |
| Imanaka2014 | 193 at-risk population | 50.2 | 14.0 | 2-arm; Japan | 3-month web-based intervention aimed at nutrition | Human-supported, remote, major, | Non eHealth intervention | Weight |
| Jahangiry2017 | 160 at-risk population | 44.0 | 27.0 | 2-arm; Iran | 3-month web-based intervention aimed at physical activity and nutrition | Self-help | Usual care | Blood pressure, glucose, cholesterol, weight |
| Javaheri2020 | 34 CVD patients | 71.6 | 25.7 | 2-arm; USA | 1.5-month web-based intervention aimed at sleep | Self-help | Waitlist | Blood pressure, sleep and relaxation |
| Johnston2016 | 174 CVD patients | 57.6 | 19.3 | 2-arm; Sweden | 6-month mobile-based intervention aimed at weight management, smoking and physical activity | Self-help | Other eHealth intervention | Cholesterol, weight, smoking |
| Keyserling2014 | 385 CVD patients | 62.0 | 48.0 | 2-arm; USA | 4-month mobile-based intervention aimed at physical activity, nutrition, and smoking | Human-supported, remote, major, professional | Usual care | Blood pressure, cholesterol, weight, cardiovascular composite score, physical activity behavior, smoking , nutrition |
| Khanji2019 | 402 CVD patients | 65.5 | 37.0 | 2-arm; The Netherlands | 6-month mobile-based intervention aimed at alcohol consumption, smoking, physical activity, weight, nutrition, stress management | Human-supported, remote only, minor, professional | Usual care | Blood pressure, glucose, cholesterol, weight |
| Kim2006 | 73 T2DM patients | N.A. | N.A. | 3-arm*; South Korea | 3-month mobile-based intervention aimed at physical activity | Human-supported, blended, minor, professional | Usual care | Glucose, physical activity behavior, alcohol |
| Kim2016 | 160 patients with hypertension, diabetes, or cardiac arrhythmia | 57.6 | 68.0 | 2-arm; South Korea | 6-month mobile-based intervention aimed at alcohol consumption, smoking and physical activity | Human-supported, remote, minor, professional | Usual care | Blood pressure, physical activity behavior, smoking, alcohol |
| Kim2019 | 151 T2DM patients | N.A. | 52.0 | 2-arm; South Korea | 2-month mobile-based intervention aimed at nutrition management, physical activity. | Human-supported, blended, major, professional | Non eHealth intervention | Glucose |
| Kirwan2013 | 72 T1DM patients | 35.2 | 61.1 | 2-arm; Australia | 4-month mobile-based intervention aimed at diet and physical activity. | Human-supported, remote, major, professional | Usual care | Glucose, nutrition |
| Kooiman2018 | 72 T2DM patients | 56.3 | N.A. | 2-arm; The Netherlands | 3-month mobile-based intervention aimed at physical activity | Human-supported, remote, minor, professional | Usual care | Glucose, weight |
| Kouwenhoven-Pasmooij2018 | 491 at risk-population | 51.0 | 19.3 | 2-arm; The Netherlands | 6-month web-based intervention, aimed depending on patient’s profile | Human-supported, blended, major, professional | Other eHealth intervention | Weight, physical activity behavior |
| Ku2020 | 40 T2DM patients | 50.0 | 65.0 | 2-arm; South Korea | 3-month web-based intervention aimed at nutrition and physical activity | Human-supported, remote, major, professional | Non eHealth intervention | Glucose, physical activity behavior, nutrition |
| Kulick2013 | 61 at-risk population | 52.2 | 75.3 | 2-arm; USA | 3-month web-based intervention aimed at nutrition | Self-help | Waitlist | Cholesterol, weight, cardiovascular composite score, nutrition |
| Kumar2020 | 300 T2DM patient | 64.65 | 60.0 | 2-arm; India | 6-month mobile-based intervention aimed at nutrition | Self-help | Usual care | Glucose |
| Lear2014 | 78 CVD patients | 60.0 | 15.0 | 2-arm; Canada | 4-month web-based intervention aimed at physical activity and nutrition | Human-supported, remote, major, professional | Usual care | Blood pressure, glucose, cholesterol, weight, physical activity capacity, physical activity behavior, smoking |
| Lee2018 | 148 T2DM patients | 52.0 | 36.5 | 2-arm; South-Korea | 6-month mobile and web-based intervention aimed at physical activity and nutrition | Human-supported, remote, major, professional | Usual care | Blood pressure cholesterol, physical activity behavior, smoking, nutrition |
| Lee2020 | 72 T2DM patients | 51.6 | 29.3 | 2-arm; South-Korea | 6-month mobile-based intervention aimed at physical activity and nutrition | Human-supported, remote, major, professional | Usual care | Glucose, cholesterol, weight, smoking, nutrition |
| Li2019 | 462 at-risk population | 61.5 | 62.8 | 2-arm; China | 6-month mobile-based intervention aimed at nutrition, physical activity, smoking, alcohol intake. | Human-supported, remote, major, layperson | Usual care | Blood pressure, glucose |
| Li2020 | 60 CKD patients | 51.3 | 26.5 | 2-arm; Taiwan | 3-month mobile-based intervention aimed at physical activity and nutrition | Human-supported, remote, major, professional | Usual care | Cholesterol, weight |
| Liebreich2009 | 49 T2DM patients | 54.1 | N.A. | 2-arm; Canada | 3-month web-based intervention aimed at physical activity | Human-supported, remote, major, professional | Other eHealth intervention | Physical activity behavior |
| Lim2021 | 204 T2DM patients | 51.2 | 35.2 | 2-arm; Singapore | 6-month mobile-based intervention aimed at physical activity and nutrition | Human-supported, remote, major, layperson | Non eHealth intervention | Blood pressure, glucose, cholesterol, weight, nutrition |
| Lindberg2017 | 166 T2DM patients | 66.6 | 29.5 | 2-arm; Sweden | 19-month web-based intervention aimed at. nutrition, physical activity, smoking and alcohol consumption | Human-supported, blended, major, professional | Usual care | Blood pressure, glucose, cholesterol |
| Lisón2020 | 105 at-risk population | 53.2 | N.A. | 2-arm; Spain | 3-month web-based intervention aimed at nutrition and physical activity | Human-supported, blended, minor, layperson | Usual care | Blood pressure, glucose, weight, physical activity capacity, physical activity behavior |
| Little2017 | 826 at-risk population | 53.7 | 63.6 | 3-arm; UK | 6-month web-based intervention aimed at nutrition and physical activity | (1) Human-supported, blended, minor, professional  (2) Human-supported, remote, minor, professional | Other eHealth intervention | Glucose, cholesterol, weight, physical activity behavior, nutrition |
| Lorig2006 | 958 CVD patients | 57.5 | 71.4 | 2-arm; USA | 1,5-month web-based intervention aimed at physical activity an stress management | Human-supported, remote, major, layperson | Usual care | Physical activity behavior, sleep |
| Lorig2010 | 761 T2DM patient | 54.3 | 72.7 | 3-arm; USA | 1,5-month web-based intervention aimed at nutrition and physical activity | (1) Human-supported, remote, major, layperson  (2) Human-supported, remote, major, layperson | Usual care | Glucose, physical activity behavior |
| Lunde2020 | 113 CVD patients | 59.0 | 22.2 | 2-arm; Norway | 12-month mobile-based intervention aimed at target behavior set by patient | Human-supported, remote, major, professional | Usual care | Blood pressure, glucose cholesterol, weight, physical activity capacity, physical activity behavior |
| Maddison2015 | 171 CVD patients | 60.2 | 19.0 | 2-arm; New Zealand | 6-month Physical activity mobile based and web-based intervention aimed at physical activity | Human-supported, remote, minor, layperson | Usual care | Physical activity capacity, physical activity behavior |
| Maddison2019 | 162 CVD patients | 61.3 | 14.2 | 2-arm; New Zealand | 3-month Physical activity mobile based and web-based intervention aimed at physical activity | Human-supported, remote, major, professional | Usual care | Blood pressure, glucose cholesterol, weight, physical activity capacity physical activity behavior |
| McDermott2018 | 200 CVD patients | 70.3 | 52.5 | 2-arm; USA | 6-month web-based intervention aimed at physical activity | Human-supported, blended, major, professional | Usual care | Physical activity capacity, physical activity behavior |
| McKay2001 | 78 T2DM patients | N.A. | N.A. | 2-arm; USA, Canada | 2-month Physical activity web-based intervention aimed at physical activity | Human-supported, remote, major, professional | Non eHealth intervention | Glucose cholesterol, nutrition |
| McKay2002 | 160 T2DM patients | 59.5 | 53.2 | 4-arm; USA | 2-month web-based intervention aimed at nutrition and others | (1) Self-help  (2) Human supported, remote, major, professional  (3) Human supported, remote, major, professional  (4) Human supported, remote, major, professional | Non eHealth intervention | Glucose cholesterol, nutrition |
| McLeod2020 | 429 T2DM and pre-diabetes patients | 62.1 | 50.9 | 2-arm; New Zealand | 13-month web-based intervention aimed at physical activity and nutrition | Human-supported, remote, major, professional | Usual care | blood pressure, glucose, weight, physical activity behavior, nutrition |
| McMahon2012 | 152 T2DM patients | 61.0 | 7.0 | 3-arm*; USA | 12-month web-based intervention aimed at physical activity and nutrition | Human-supported, remote, major, professional | Non eHealth intervention | Glucose, cholesterol, weight |
| Mensorio2019 | 106 at-risk population | N.A. | N.A. | 2-arm; Spain | 3-month web-based intervention aimed at physical activity and nutrition | Self-help | Usual care | Weight, physical activity behavior |
| Murray2018 | 374 T2DM patients | 64.8 | 71.0 | 2-arm; UK | 12-month web-based interventions aimed at physical activity, nutrition, smoking, alcohol intake. | Self-help | Other eHealth intervention | Blood pressure, glucose |
| Nolan2018 | 264 at-risk population | 57.6 | 58.5 | 2-arm; Canada | 3-month Web-based intervention aimed at nutrition, smoking and physical activity | Self-help | Other eHealth intervention | Blood pressure, cholesterol, cardiovascular composite score |
| Orsama2013 | 56 T2DM patients | 61.9 | 46.0 | 2-arm; Finland | 10-month Web-based and mobile-based intervention aimed at nutrition and physical activity | Human-supported, remote, minor, professional | Usual care | Blood pressure glucose, weight, |
| Paldán2021 | 47 CVD patients | 65.1 | 46.0 | 2-arm; Germany | 6-month mobile-based intervention aimed at physical activity | Self-help | Usual care | Physical activity capacity, physical activity behavior |
| Park2021 | 60 CVD patients | 66.8 | 21.5 | 2-arm; USA | 2-month mobile-based intervention aimed at physical activity | Human-supported, remote, major, layperson | Non eHealth intervention | Physical activity capacity, physical activity behavior |
| Peacock2020 | 204 at-risk population | 63.5 | 36.0 | 2-arm; UK | 3-month Web-based intervention aimed at physical activity | Human-supported, blended, major, professional | Non eHealth intervention | Blood pressure, glucose, cholesterol, weight, physical activity behavior |
| Plotnikoff2017 | 84 T2DM and at-risk patients | 44.7 | 70.2 | 2-arm; Australia | 5-month web-based intervention aimed at physical activity | Human-supported, blended, major, professional | Waitlist | Blood pressure, weight, physical activity capacity, physical activity behavior |
| Quinn2011 | 213 T2DM patients | 52.6 | 50.0 | 4-arm*; USA | 12-month web-based and mobile-based intervention aimed at physical activity and nutrition | Self-help | Usual care | Blood pressure, glucose, cholesterol |
| Riangkam2021 | 129 T1DM patients | 51.5 | 39.6 | 3-arm; Thailand | 3-month mobile-based intervention aimed at | Human-supported, blended, major, layperson | Usual care | Glucose |
| Richard2019 | 2724 CVD, diabetes or at-risk patients | 69.0 | 47.7 | 2-arm; The Netherlands, Finland, France | 18-month web-based intervention aimed at nutrition, smoking and physical activity | Human-supported, remote, minor, professional | Other eHealth intervention | Cholesterol, cardiovascular composite score, physical activity capacity, physical activity behavior, smoking, nutrition |
| Siebmanns2021 | 48 CVK patients | 72.5 | 35.5 | 2-arm; Sweden | 2-month web-based intervention aimed at sleep | Human-supported, remote, major, professional | Other eHealth intervention | Sleep |
| Skobel2017 | 118 CVD patients | 59.0 | 11.0 | 2-arm; Spain, Germany, UK | 6-month web and mobile-based intervention aimed at physical activity | Human-supported, remote, minor, professional | Usual care | Cholesterol, physical activity capacity |
| Smith2009 | 41 at-risk population | 43.5 | N.A. | 2-arm; USA | 4-month web-based intervention aimed at physical activity | Human-supported, remote, minor, layperson | Waitlist | Blood pressure, glucose, insulin, cholesterol, weight, cardiovascular composite score, physical activity capacity, physical activity behavior |
| Spring2017 | 96 at-risk population | 40.2 | 86.0 | 3-arm; USA | 6-month mobile-based intervention aimed at nutrition and physical activity | Human-supported, blended, major, professional | Non eHealth intervention | Weight |
| Su2021 | 146 CVD patients | 55.8 | 32.9 | 2-arm; China | 3-month mobile-based intervention aimed at nutrition, stress, smoking | Human-supported, remote, minor, professional | Usual care | Blood pressure, glucose, weight, physical activity behavior |
| Tanaka2018 | 112 at-risk population | 46.7 | 0.7 | 2-arm; Japan | 2-month mobile-based intervention aimed at nutrition | Human-supported, remote, major, professional | Waitlist | Blood pressure, cholesterol, weight, nutrition |
| Tang2013 | 415 T2DM patients | 53.8 | 40.1 | 2-arm; USA | 12-month Web-based intervention aimed at physical activity and nutrition | Human-supported, remote, major, layperson | Usual care | Blood pressure, glucose, cholesterol, weight, cardiovascular composite score |
| Thomas2015 | 154 at-risk population | 53.2 | 79.9 | 2-arm; USA | 3-month web-based intervention aimed at physical activity | Human-supported, blended, minor, professional | Other eHealth intervention | Blood pressure, glucose, weight |
| Tjam2006 | 57 T1DM and T2DM patients | N.A. | 53.2 | 2-arm; USA | 12-month Web-based intervention aimed at | Human-supported, major, remote, professional | Usual care | Glucose, Cholesterol |
| Tomita2009 | 40 CVD patients | 75.9 | 68.8 | 2-arm; USA | 12-month web-based intervention aimed at | Human-supported, minor, professional | Usual care | Blood pressure, sleep |
| Turnin2021 | 282 T2DM patients | 59.6 | 36.9 | 2-arm; France | 12-month web-based and mobile-based intervention aimed at physical activity and nutrition | Self-help | Usual care | Glucose |
| Van der Weegen2013 | 199 T1DM and T2DM patients | 58.4 | 53.4 | 3-arm; The Netherlands | 6-month web-based and mobile-based intervention | Human-supported, major, blended, professional | Usual care | Physical activity behavior |
| Vluggen2021 | 478 T2DM patients | 60.2 | 32.5 | 2-arm; The Netherlands | 6-month mobile-based intervention aimed at physical activity and nutrition | Self-help | Waitlist | Physical activity behavior |
| Vogel2017 | 36 CVD patients | 62.8 | 0 | 2-arm; Austria | 1.5-month mobile-based intervention aimed at physical activity | Self-help | Usual care | Physical activity capacity |
| Watson2015 | 65 at-risk population | 52.2 | 55.5 | 2-arm; UK | 12-month web-based intervention aimed at nutrition and physical activity | Self-help | Usual care | Blood pressure, cholesterol, weight, nutrition |
| Wayne2015 | 131 T2DM patients | 53.2 | 72.5 | 2-arm; Canada | 6-month mobile-based intervention aimed at nutrition and physical activity | Human-supported, blended, major, professional | Non eHealth intervention | Glucose, weight |
| Widmer2017 | 80 CVD patients | 63.1 | 18.5 | 2-arm; USA | 3-month mobile- and web-based intervention aimed at physical activity, nutrition, and smoking | Self-help | Usual care | Blood pressure, glucose, cholesterol, weight, physical activity behavior, nutrition |
| Widyanata2019 | 65 T2DM patients | N.A. | 56.7 | 2-arm; Indonesia | 3-month mobile-based intervention aimed at nutrition and physical activity | Self-help | Waitlist | Glucose, insulin cholesterol, |
| Wong2020 | 438 CVD patients | 52.4 | 34.5 | 2-arm; China | 6-month web-based intervention aimed at physical activity | Self-help | Usual care | Blood pressure, cholesterol physical activity behavior |
| Wong2021 | 77 at-risk population | 59.0 | 55.8 | 2-arm; China | 3-month mobile-based intervention aimed at physical activity, stress and nutrition | Self-help | Non eHealth intervention | Blood pressure, glucose, cholesterol, weight, physical activity behavior |
| Wongrochananan2015 | 126 T2DM patients | 52.5 | 46.8 | 2-arm; Thailand | 3-month web-based intervention aimed at physical activity, and nutrition. | Self-help | Usual care | Blood pressure, glucose, cholesterol, weight, nutrition |
| Yu2019 | 92 T2DM patients | 52.3 | 29.7 | 2-arm; China | 6-month mobile-based intervention aimed at nutrition and physical activity | Human-supported, remote, major, professional | Usual care | Blood pressure, glucose, weight |
| Yudi2021 | 206 CKD patients | 56.5 | 31.2 | 2-arm; USA | 2-month mobile-based intervention aimed at physical activity | Human-supported, remote, minor, professional | Usual care | Glucose, cholesterol, weight, physical activity behavior, smoking |
| Zhang2019 | 156 T1DM and T2DM patients | 53.5 | 39.1 | 2-arm; China | 6-month mobile-based intervention aimed at nutrition and physical activity | Human-supported, remote, major, professional | Usual care | Glucose, cholesterol, weight |

*multiple control conditions, only the least extensive condition was extracted

CVD = cardiovascular disease; CKD = chronic kidney disease; T1DM = type 1 diabetes mellitus; T2DM = type 2 diabetes mellitus

**Table S2.** Risk of bias assessment of the included studies.

| **Study** | **D1** | **D2** | **D3** | **D4** | **D5** | **Overall** |
| --- | --- | --- | --- | --- | --- | --- |
| Agarwal2019(a) | **!** | **!** | **+** | **+** | **+** | **!** |
| Agarwal2019(b) | **!** | **!** | **+** | **+** | **+** | **!** |
| Akinci2019 | **+** | **+** | **+** | **+** | **+** | **+** |
| Appel2011 | **!** | **-** | **-** | **!** | **+** | **-** |
| Baer2020 | **+** | **+** | **!** | **+** | **+** | **!** |
| Bailey2020 | **!** | **!** | **+** | **+** | **+** | **!** |
| Bennett2010 | **+** | **+** | **+** | **+** | **!** | **!** |
| Bennett2018 | **+** | **+** | **+** | **+** | **!** | **!** |
| Bond2007 | **!** | **!** | **+** | **+** | **!** | **!** |
| Bozorgi2021 | **+** | **!** | **+** | **!** | **!** | **!** |
| Cai2021 | **!** | **!** | **+** | **+** | **!** | **!** |
| Chao2019 | **!** | **!** | **+** | **-** | **!** | **-** |
| Choi2019 | **!** | **+** | **+** | **+** | **+** | **!** |
| Chow2020 | **!** | **!** | **+** | **+** | **!** | **!** |
| Connely2017 | **+** | **!** | **+** | **+** | **+** | **!** |
| Dorje2019 | **+** | **+** | **+** | **+** | **+** | **+** |
| Dorsch2020 | **!** | **!** | **+** | **!** | **+** | **!** |
| Duan2018 | **!** | **!** | **+** | **!** | **!** | **!** |
| Duscha2018 | **!** | **+** | **+** | **+** | **!** | **!** |
| Engelen2020 | **+** | **+** | **-** | **!** | **!** | **-** |
| Fukuoka2015 | **+** | **!** | **+** | **!** | **!** | **!** |
| Glasgow2012 | **+** | **+** | **+** | **!** | **!** | **!** |
| Gong2020 | **+** | **+** | **+** | **+** | **!** | **!** |
| Grau-Pellicer2020 | **!** | **!** | **+** | **!** | **!** | **!** |
| Gunawardena2019 | **+** | **+** | **!** | **+** | **!** | **!** |
| Hanswel2017 | **!** | **+** | **+** | **+** | **!** | **!** |
| Haste2017 | **+** | **!** | **!** | **+** | **!** | **!** |
| Hilmarsdóttir2021 | **+** | **+** | **+** | **+** | **!** | **!** |
| Höchsmann2019 | **+** | **+** | **+** | **+** | **+** | **+** |
| Holmen2014 | **+** | **+** | **+** | **+** | **+** | **+** |
| Houchen-Wolloff2018 | **+** | **!** | **!** | **+** | **!** | **!** |
| Humalda2020 | **+** | **+** | **+** | **+** | **!** | **!** |
| Imanaka2014 | **!** | **+** | **+** | **+** | **!** | **!** |
| Jahangiry2015 | **!** | **+** | **-** | **+** | **!** | **-** |
| Javaheri2020 | **!** | **!** | **+** | **+** | **+** | **!** |
| Johnston2016 | **!** | **!** | **+** | **+** | **+** | **!** |
| Keyserling2014 | **-** | **!** | **+** | **!** | **!** | **-** |
| Khanji2019 | **+** | **+** | **+** | **+** | **+** | **+** |
| Kim2006 | **!** | **!** | **+** | **+** | **+** | **!** |
| Kim2016 | **+** | **!** | **+** | **!** | **+** | **!** |
| Kim2019 | **+** | **!** | **!** | **+** | **!** | **!** |
| Kirwan2013 | **!** | **!** | **+** | **+** | **!** | **!** |
| Kooiman2018 | **!** | **+** | **+** | **+** | **!** | **!** |
| Kouwenhoven-Pasmooij2018 | **+** | **+** | **+** | **!** | **-** | **-** |
| Ku2020 | **!** | **!** | **+** | **+** | **!** | **!** |
| Kulick2013 | **+** | **!** | **+** | **!** | **!** | **!** |
| Kumar2020 | **+** | **!** | **+** | **+** | **!** | **!** |
| Lear2014 | **+** | **+** | **+** | **+** | **!** | **!** |
| Lee2018 | **+** | **!** | **+** | **!** | **!** | **!** |
| Lee2020 | **+** | **!** | **+** | **!** | **!** | **!** |
| Li2019 | **-** | **!** | **+** | **+** | **+** | **-** |
| Li2020 | **!** | **!** | **+** | **+** | **!** | **!** |
| Liebreich2009 | **!** | **+** | **+** | **!** | **!** | **!** |
| Lim2021 | **+** | **+** | **+** | **+** | **+** | **+** |
| Lindberg2017 | **+** | **+** | **-** | **+** | **-** | **-** |
| Lisón2020 | **+** | **+** | **!** | **+** | **!** | **!** |
| Little2017 | **+** | **+** | **+** | **+** | **!** | **!** |
| Lorig2006 | **!** | **!** | **+** | **!** | **!** | **!** |
| Lorig2010 | **!** | **+** | **+** | **!** | **!** | **!** |
| Lunde2020 | **+** | **!** | **+** | **+** | **!** | **!** |
| Maddison2015 | **+** | **+** | **!** | **!** | **+** | **!** |
| Maddison2019 | **+** | **+** | **+** | **+** | **+** | **+** |
| McDermott2018 | **!** | **+** | **+** | **+** | **+** | **!** |
| McKay2001 | **!** | **!** | **+** | **!** | **!** | **!** |
| McKay2002 | **!** | **!** | **+** | **!** | **!** | **!** |
| McLeod2020 | **+** | **+** | **+** | **+** | **+** | **+** |
| McMahon2012 | **+** | **+** | **+** | **+** | **!** | **!** |
| Mensorio2019 | **+** | **+** | **-** | **+** | **+** | **-** |
| Murray2018 | **!** | **+** | **+** | **+** | **!** | **!** |
| Nolan2018 | **+** | **+** | **!** | **+** | **!** | **!** |
| Orsama2013 | **!** | **!** | **!** | **+** | **!** | **!** |
| Paldán2021 | **!** | **!** | **+** | **+** | **+** | **!** |
| Park2021 | **!** | **!** | **!** | **+** | **!** | **!** |
| Peacock2020 | **+** | **+** | **+** | **+** | **+** | **+** |
| Plotnikoff2017 | **+** | **+** | **!** | **+** | **+** | **!** |
| Quinn2011 | **!** | **+** | **-** | **+** | **+** | **-** |
| Riangkam2021 | **+** | **+** | **+** | **+** | **!** | **!** |
| Richard2019 | **!** | **+** | **+** | **+** | **+** | **!** |
| Siebmanns2021 | **!** | **+** | **!** | **!** | **!** | **!** |
| Skobel2017 | **!** | **!** | **-** | **+** | **!** | **-** |
| Smith2009 | **!** | **+** | **+** | **+** | **!** | **!** |
| Spring2017 | **+** | **+** | **-** | **+** | **!** | **-** |
| Su2021 | **+** | **!** | **+** | **+** | **+** | **!** |
| Tanaka2018 | **!** | **+** | **+** | **+** | **!** | **!** |
| Tang2013 | **!** | **+** | **+** | **+** | **!** | **!** |
| Thomas2015 | **+** | **+** | **+** | **!** | **!** | **!** |
| Tjam2006 | **!** | **!** | **!** | **+** | **!** | **!** |
| Tomita2009 | **!** | **+** | **+** | **!** | **!** | **!** |
| Turnin2021 | **!** | **+** | **+** | **+** | **!** | **!** |
| Van der Weegen2013 | **+** | **+** | **!** | **+** | **+** | **!** |
| Vluggen2021 | **+** | **+** | **+** | **!** | **+** | **!** |
| Vogel2017 | **+** | **+** | **+** | **+** | **!** | **!** |
| Watson2015 | **+** | **+** | **+** | **+** | **!** | **!** |
| Wayne2015 | **+** | **+** | **+** | **+** | **!** | **!** |
| Widmer2017 | **!** | **!** | **!** | **+** | **!** | **!** |
| Widyanata2019 | **!** | **!** | **-** | **+** | **!** | **-** |
| Wong2020 | **!** | **+** | **+** | **+** | **!** | **!** |
| Wong2021 | **+** | **+** | **+** | **+** | **!** | **!** |
| Wongrochananan2015 | **!** | **+** | **!** | **!** | **!** | **!** |
| Yu2019 | **+** | **+** | **+** | **+** | **!** | **!** |
| Yudi2021 | **+** | **+** | **+** | **+** | **+** | **+** |
| Zhang2019 | **+** | **+** | **+** | **+** | **!** | **!** |

Domain 1: Risk of bias arising from the randomization process

Domain 2: Risk of bias due to deviations from the intended interventions

Domain 3: Risk of bias due to missing outcome data

Domain 4: Risk of bias in measurement of the outcome

Domain 5: Risk of bias in selection of the reported result

| **+** | Low risk |
| --- | --- |
| **!** | Some concerns |
| **-** | High risk |
